# Clustering benchmark datasets exploiting the fundamental clustering problems

**DOI:** 10.1016/j.dib.2020.105501

**Published:** 2020-04-20

**Authors:** Michael C. Thrun, Alfred Ultsch

**Affiliations:** a Databionics Research Group, Philipps-University of Marburg, Hans-Meerwein-Straße 6, D-35032 Marburg, Germany; b Dept. of Hematology, Oncology and Immunology, Philipps-University Marburg, Germany

**Keywords:** Cluster analysis, Dimensionality reduction, Pattern recognition, Projection methods

## Abstract

The Fundamental Clustering Problems Suite (FCPS) offers a variety of clustering challenges that any algorithm should be able to handle given real-world data. The FCPS consists of datasets with known a priori classifications that are to be reproduced by the algorithm. The datasets are intentionally created to be visualized in two or three dimensions under the hypothesis that objects can be grouped unambiguously by the human eye. Each dataset represents a certain problem that can be solved by known clustering algorithms with varying success. In the R package “Fundamental Clustering Problems Suite” on CRAN, user-defined sample sizes can be drawn for the FCPS. Additionally, the distances of two high-dimensional datasets called Leukemia and Tetragonula are provided here. This collection is useful for investigating the shortcomings of clustering algorithms and the limitations of dimensionality reduction methods in the case of three-dimensional or higher datasets. This article is a simultaneous co-submission with Swarm Intelligence for Self-Organized Clustering [1].

Specifications table

**Table:** 

Subject	Computer Science
Specific subject area	Unsupervised Machine Learning
Type of data	All files are ASCII text files. TAB separates columns. Headers are included. *.lrn files contain the data, including a unique key for each case; *.cls contain keys and class labels. A positive number indicates each class. For Tetragonula, the geographic coordinates are included as a separate *.lrn.
How data were acquired	Artificially, except for the two high-dimensional datasets Leukemia and Tetragonula. In this case, the distance matrices and Databionic swarm clusterings are included.
Data format	FCPS: Raw; High-dimensional datasets: Preprocessed.
Parameters for data collection	For artificial datasets, none; for High-Dimensional datasets, please see below.
Description of data collection	For artificial datasets none; for Leukemia and Tetragonula, please see below.
Data source location	For artificial datasets none; for Leukemia and Tetragonula, please see below.
Data accessibility	FCPS In R: https://CRAN.R-project.org/package=FCPS Complete data attached to this article.
Related research article	Co-submission of the revision of M. C. Thrun, and A. Ultsch, “Swarm Intelligence for Self-Organized Clustering,” Journal of Artificial Intelligence, in press, DOI: 10.1016/j.artint.2020.103237, 8. Jan, 2020.

## Value of the data

• FCPS is a collection of intentionally low-dimensional artificial datasets of user-defined sample sizes and an unique class labeling generated under the hypothesis that humans are most often able to group objects in two- or three-dimensional plots by eye.
• FCPS offers a variety of real-world challenges, such as outliers or density vs. distance-defined clusters, on which the performance of clustering algorithms can be tested.
• Additionally, two high-dimensional real-world datasets with a clear cluster structure are provided:
  ○ Any clustering of the Tetragonula dataset should be coherent with the geographic locations not used in the clustering, and the dataset presents the challenges that density information cannot be used directly, and the existence of many clusters and several outliers.
  ○ The Leukemia dataset possesses high-dimensional cluster structures that are consistent with the unambiguously defined diagnosis of patients of unbalanced class sizes.

## 1 Data

This work presents a specific collection of twelve datasets with easy access via the programming language R or attached to this work. In [1], these datasets were used to benchmark several clustering methods. The collection consists of two real-world examples of high-dimensional datasets and ten artificial datasets. Each dataset has a specific clustering challenge, which is summarized in Table 1. Lsun3D and each of the nine artificial datasets of the formerly Fundamental Clustering Problems Suite (FCPS) were defined separately for a specific clustering problem, as cited below, but nine of the artificial datasets presented here were named FCPS by Ultsch in 2005 in [2]. The original sample sizes defined in the respective first publications mentioning the datasets were used in [1], but the R function “ClusterChallenge” of the FCPS package on CRAN (https://CRAN.R-project.org/package=FCPS) can be used to draw a sample of 300 or more for all artificial datasets. Additionally, the ability to preserve the cluster structures of two-dimensional projections after dimensionality reduction can be investigated in the case of eight datasets that have a dimensionality of three or higher.

**Table 1:** Summary of the description and challenges of the 12 datasets for cluster analysis, and in case of not 2D datasets for projection methods.

Name of Dataset	Short Description of Shape or Content	Challenge
Atom	Core enclosed by hull	Completely overlapping convex hull
Chainlink	Two intertwined chains	Linear nonseparable entanglements
EngyTime	Two Gaussian mixtures with different variance	Overlapping clusters separable only by density
GolfBall	Empty sphere	No distance-based cluster structures
Hepta	Six balls, each centered at each one of the six corners of a large octahedron with the 7th ball having a higher density at its center	Nonoverlapping convex hulls with varying intracluster distances
Lsun3D	One full sphere, two bricks at a perpendicular angle to each other, and outliers	Varying geometric shapes with noise defined by one group of outliers
Target	Circular disk enclosed by a circle with outliers in four corners	Overlapping convex hulls combined with noise defined by four groups of outliers
Tetra	Four close full spheres at the four corners of a tetrahedron	Narrow distances between the clusters
TwoDiamonds	Two rhombs with one touching corner	Identification of the weak link in chain-like connected clusters
WingNut	Two rectangles, each having a density that increases towards one corner in the direction of the other rectangle	Short intercluster distances combined with vast intracluster distances
Tetragonula	Distance matrix easy associable with geographic origins of cases	Smooth transition between clusters and outliers, clusters have to be coherent with geographic origins
Leukemia	Distance matrix easy associable with patient diagnosis of cases	Reproducing highly unbalanced class sizes

### 1.1 Atom

**Fig. 1:**
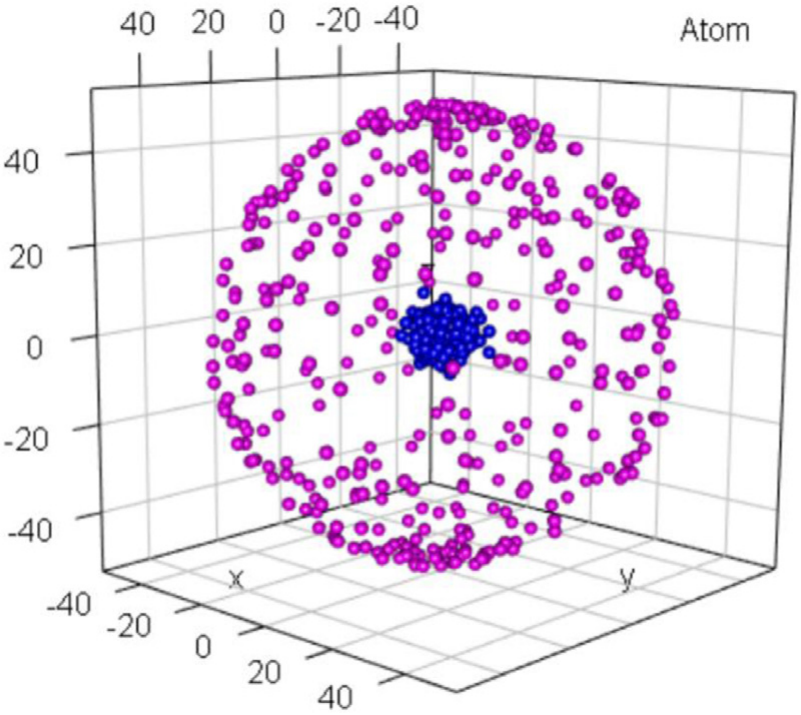
Visualization of the Atom dataset of a core enclosed by a hull. The predefined classification is indicated by color.

The Atom dataset, which was defined in [3] and is shown in Fig. 1, consists of two clusters in $R^{3}$ with a completely overlapping convex hull. In Cartesian metric space, Atom is specifically defined to be linearly nonseparable because the first cluster entirely encloses the second one. The second cluster of the core, initially with 400 points, is located in the center and surrounded by a well-separated cluster of the hull with 400 initial points [3]. Moreover, the density of the core is larger than the density in the hull by several orders of magnitude [3]. “The inner cluster variance of the hull points is also larger than the distances between the clusters” [3].

### 1.2 Chainlink

**Fig. 2:**
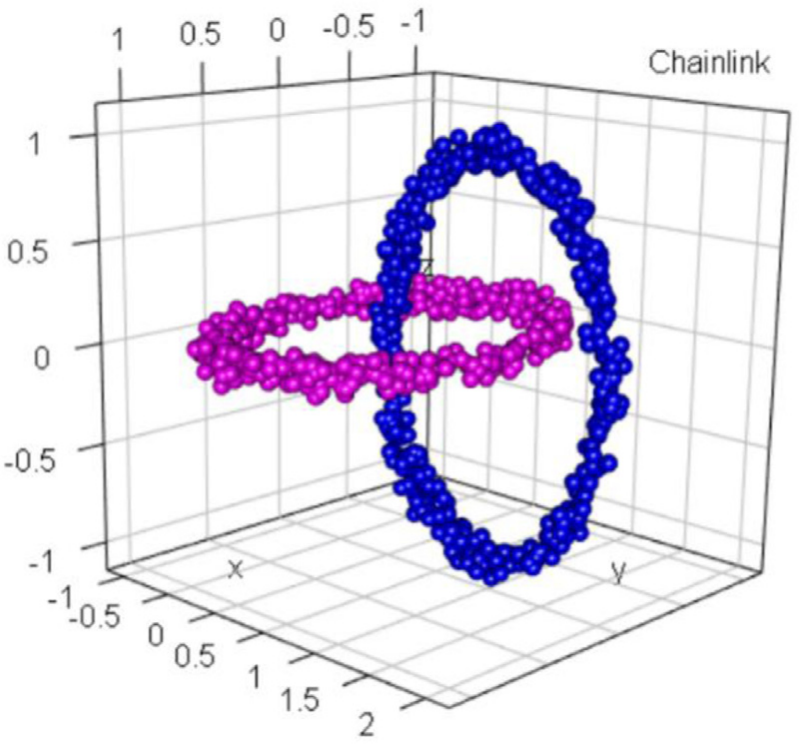
Visualization of the Chainlink dataset of two intertwined chains. The predefined classification is indicated by color.

The Chainlink dataset, which was defined in [4,5], consists of two clusters, as shown in Fig. 2. Every cluster initially contains 500 points [4,5]. Together, the two clusters form intricate links of a chain, presenting the problem of linear nonseparable entanglement. The rings are cohesive in $R^{3}$. This dataset serves as an excellent demonstration of several challenges. The data lie on two well-separated manifolds such that the global proximities contradict the local ones in the sense that the center of each ring is closer to some elements of the other cluster than to elements of its own cluster [6]. The two rings are intertwined in $R^{3}$; furthermore, they have the same average distances and densities.

### 1.3 EngyTime

**Fig. 3:**
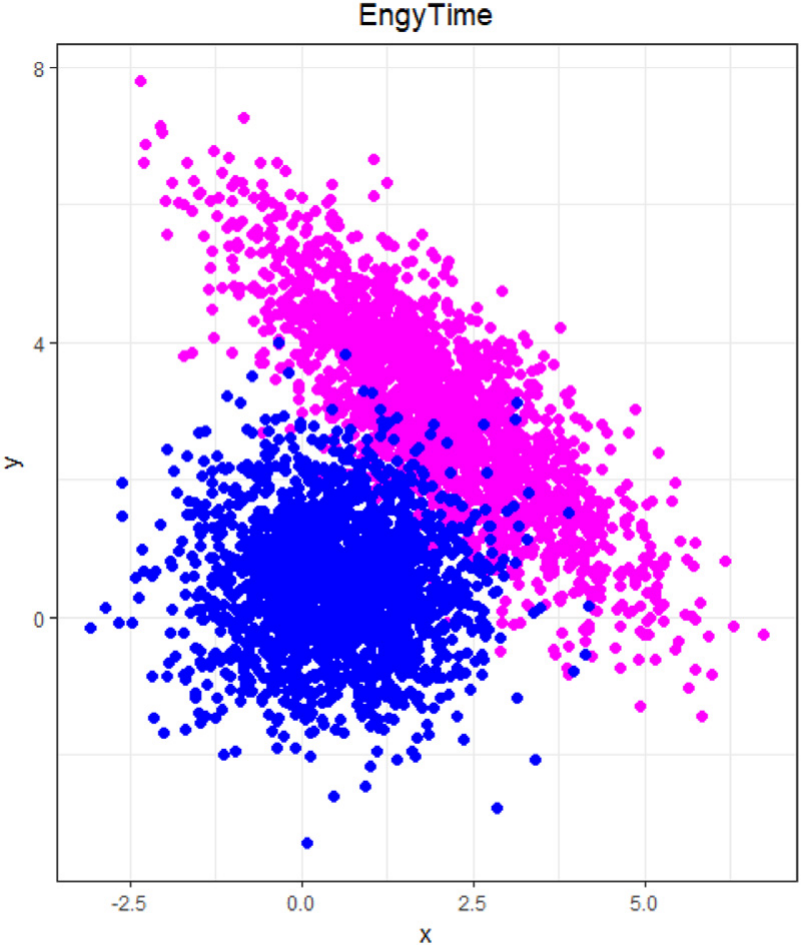
Visualization of the EngyTime dataset of two Gaussian mixtures with different variance. The predefined classification is indicated by color.

The EngyTime dataset, which was published in [7] and is shown in Fig. 3, initially contains 4096 points belonging to two clusters in $R^{2}$. The dataset serves as a simpliciation of a common density problem as presented, for example, in unclassified high-dimensional flow cytometry data [8]. EngyTime is a two-dimensional mixture of Gaussian distributions, typical of sonar applications with the variables “Engy” and “Time”. The clusters overlap, and the cluster borders can only be defined using density information because there is no empty space between clusters.

### 1.4 GolfBall

**Fig. 4:**
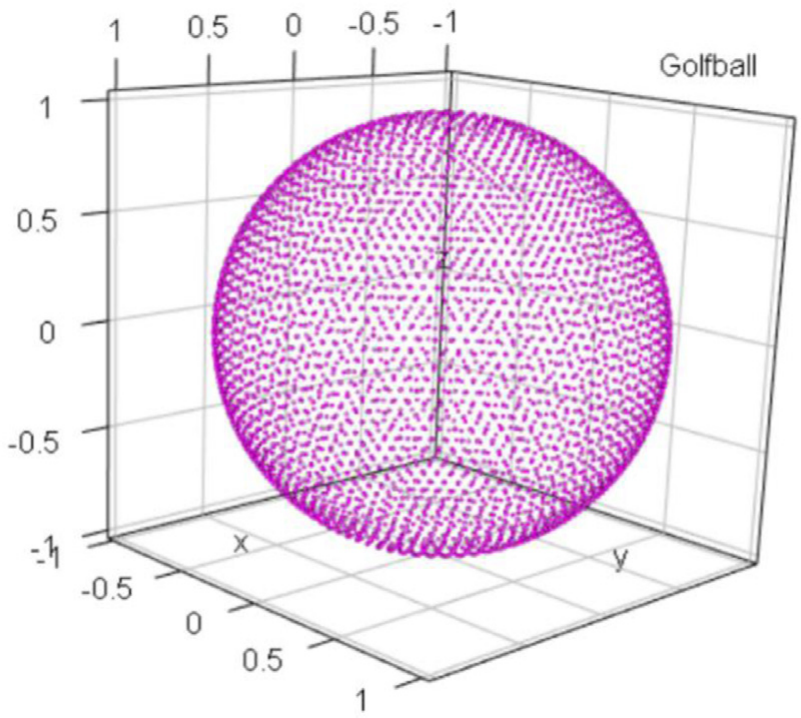
Visualization of the GolfBall dataset of an empty sphere. The predefined classification is indicated by color.

The GolfBall dataset is shown in Fig. 4, consists of an artificial dataset with 4002 points in [2], resembling a 3D view of a golf ball [9] in $R^{3}$. Originally, the points were located on the surface of a sphere at equal distances from each of the six nearest neighbors [9]. Although the dataset is based on the relative relationship between data points and the dataset can be partitioned by dividing the sphere into parts, no distance-based cluster structures exist because the range of intracluster distances can never be smaller than the range of intercluster distances.

### 1.5 Hepta

**Fig. 5:**
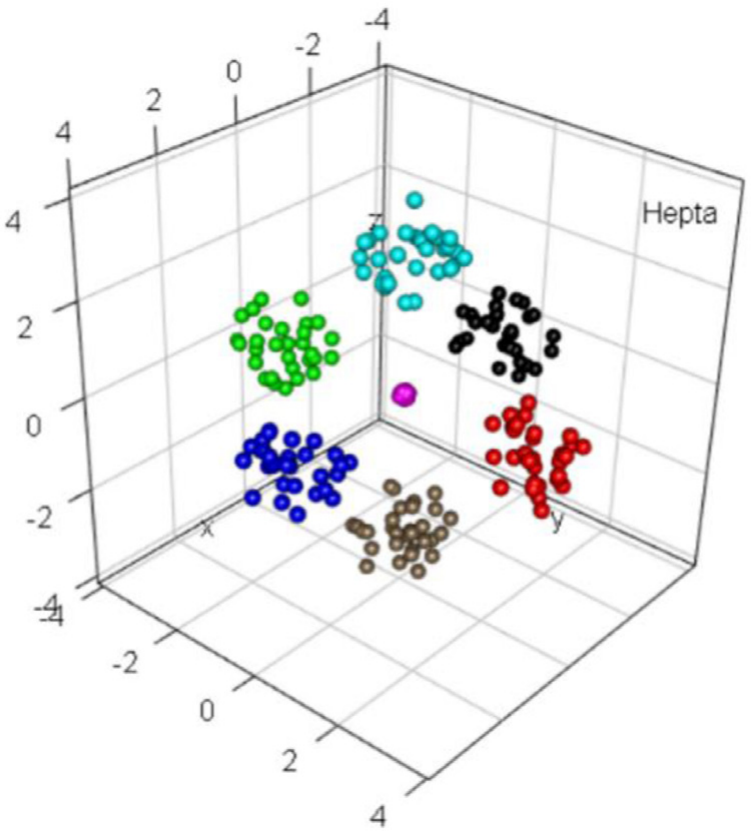
Visualization of the Hepta dataset of six balls with their centers at the six corners of a large octahedron and a 7th ball with a higher density at the center in magenta. The predefined classification is indicate by color.

The 3D Hepta dataset, which was defined in [10], consists of seven clusters that are clearly separated by distances. The seventh cluster in the center has a substantially higher density (depicted in magenta in Fig. 5). The challenge of Hepta is the nonoverlapping convex hulls with varying intracluster distances. Originally, the dataset consisted of 212 points, comprising seven clusters of thirty points each plus two additional points in the center cluster. The centroids of the clusters span the coordinate axes of $R^{3}$. The density of the central cluster is almost twice as high as the density of the other six clusters.

### 1.6 Lsun3D

**Fig. 6:**
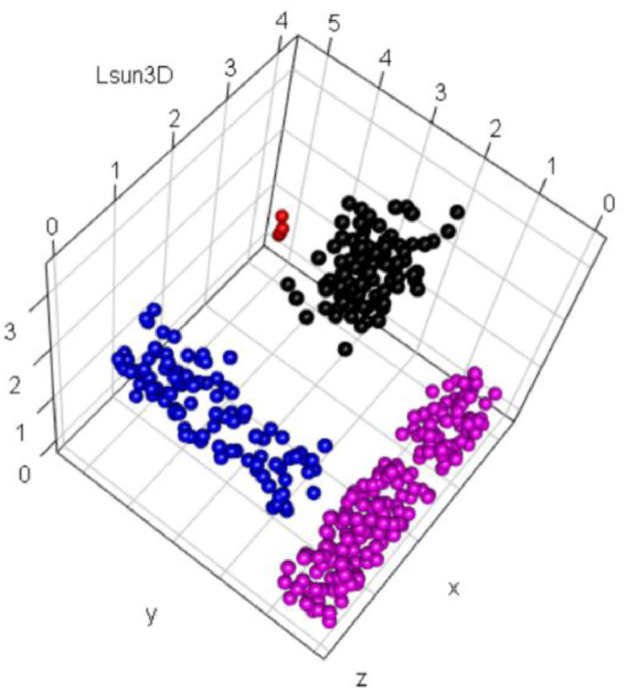
Visualization of the Lsun3D dataset of one full sphere, two bricks at perpendicular angle to each other, and outliers in red. The predefined classification is indicated by color.

The Lsun3D dataset shown in Fig. 6 consists of three well-separated clusters and four outliers in $R^{3}$ and was originally published in [11]. Lsun3D is based on the two-dimensional Lsun dataset of [1]. The challenge of Lsun3D is the nonoverlapping convex hulls with varying geometric shapes with noise defined by one small group of outliers. Two of the clusters originally contained 100 points each, and the third contained 200 points. The intercluster minimum distances, however, are in the same range as or smaller than the intracluster mean distances [12]. The dataset consists of 404 data points.

### 1.7 Target

**Fig. 7:**
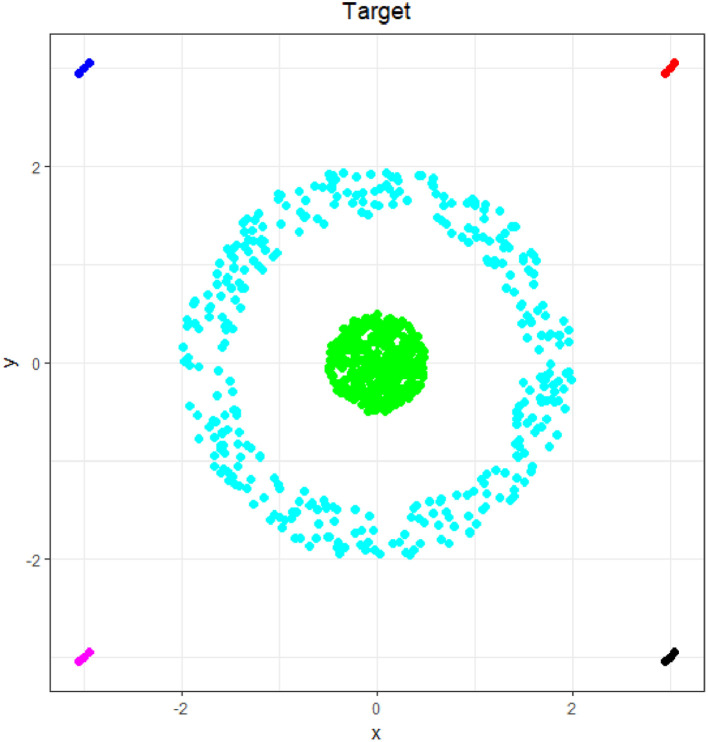
Visualization of the Target dataset of a circular disk enclosed by a circle with outliers in four corners. The predefined classification is indicated by color.

The Target dataset, which was defined in [13], is shown in Fig. 7 and consists of two main clusters and four groups of four outliers each in $R^{2}$. The first main cluster is a sphere of (formerly) 365 points, and the second cluster is a ring around the sphere consisting of 395 points. The dataset as a whole consists of 770 points in $R^{2}$. The main challenge of this dataset is the overlapping convex hulls combined with noise defined by the four small groups of outliers in the four corners.

### 1.8 Tetra

**Fig. 8:**
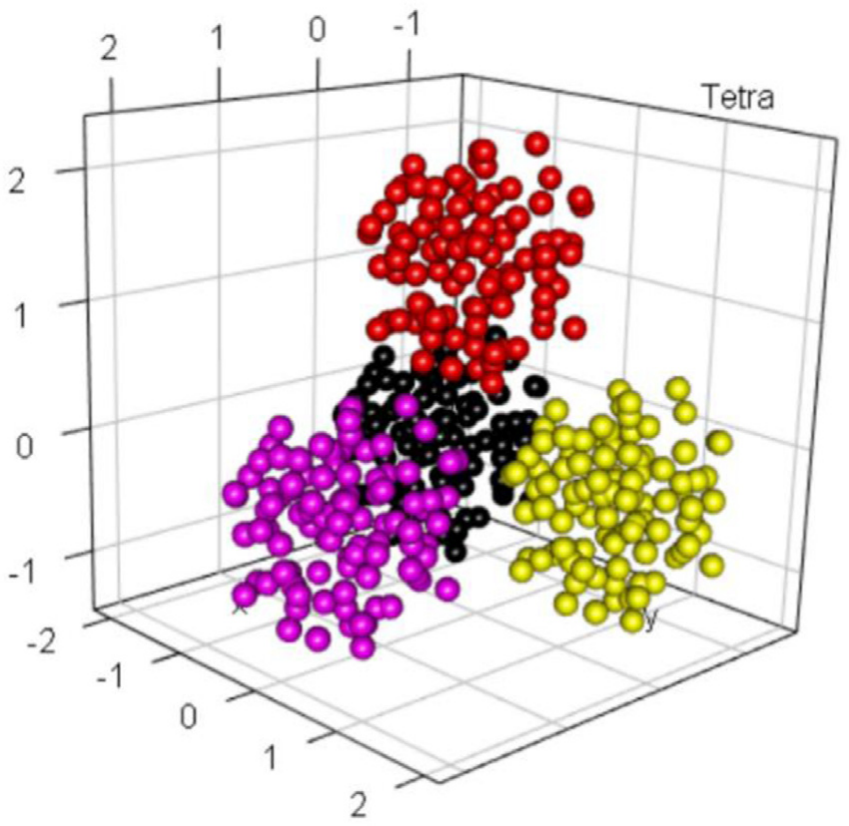
Visualization of the Tetra dataset of four large full spheres close to each other centering at the four corners of a tetrahedron. The predefined classification is indicated by color.

The Tetra dataset was defined in [14,15] and is shown in Fig. 8. The dataset originally consisted of 400 data points in four spherical clusters in $R^{3}$ that have large intracluster distances [13]. The clusters nearly touch each other, resulting in the challenge of low intercluster distances.

### 1.9 TwoDiamonds

**Fig. 9:**
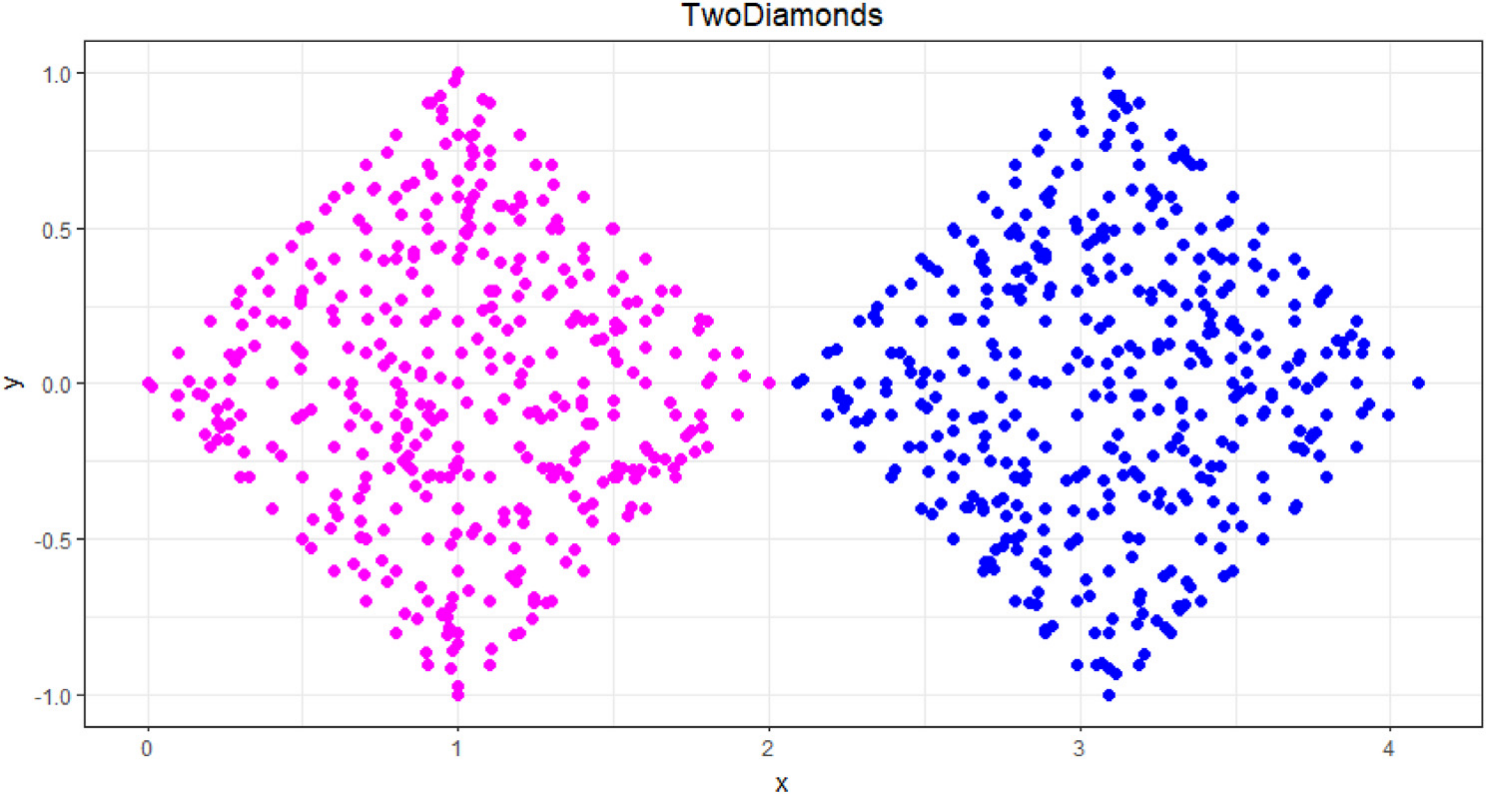
Visualization of the TwoDiamonds dataset of two rhombs with one touching corner. The predefined classification is indicated by color.

The TwoDiamonds dataset, which was defined in [16,17], is shown in Fig. 9 and consists of two clusters of two-dimensional points. “Inside each ‘diamond’, the values for each data point were drawn independently from uniform distributions” [16]. The clusters originally contained 300 points each. “[In] [e]ach cluster[, the] points are uniformly distributed within a square, and at one point the two squares almost touch” [12]. This dataset is challenging for clustering algorithms that use only distance because the clusters are connected like a chain, making it difficult to identify the weak link.

### 1.10 WingNut

**Fig. 10:**
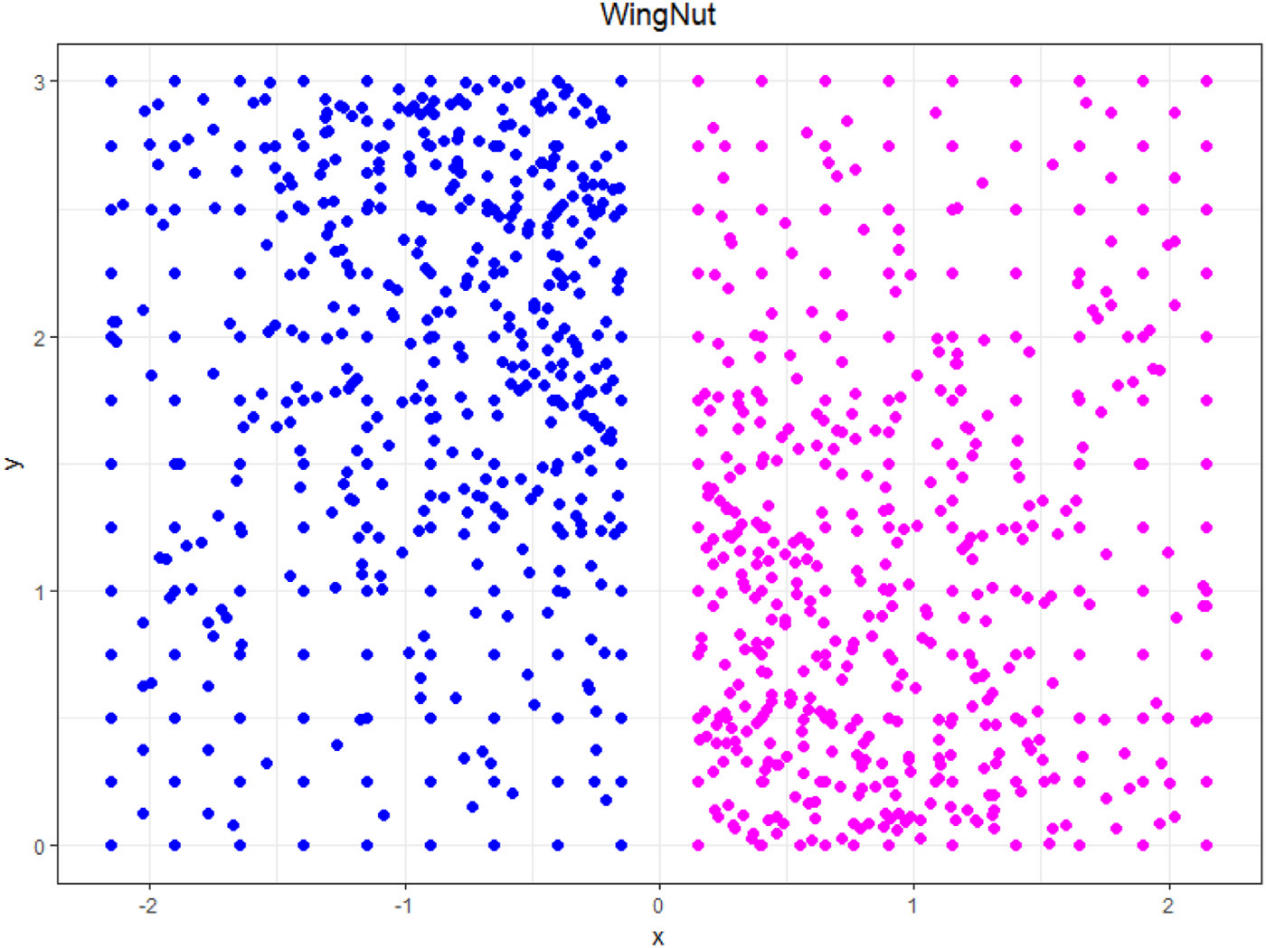
Visualization of the WingNut dataset of two rectangles, each having a density that increases in direction of the other rectangle towards one corner. The predefined classification is indicated by color.

The WingNut dataset shown in Fig. 10 consists of two symmetric data subsets originally of 500 points each [2]. “Each of these subsets is an overlay of equal[ly] spaced points with a lattice distance of 0.2 and random points with a growing density in one corner. The data sets are mirrored and shifted such that the gap between the subsets is larger than 0.3. There is a bigger distance between the subsets than within the data of a subset” [12]. This dataset is challenging for clustering algorithms that use only distance because of the small intercluster distance relative to the large intracluster distance.

### 1.11 Tetragonula

The Tetragonula dataset was published in [18]. For this dataset, clustering must be based on only a distance matrix, and any clustering must be coherent with an external validation of geographic origins. The clustering challenge is the smooth transition between clusters and outliers. Clusters should have smaller intracluster than intercluster distances while remaining coherent with the geographic origins.

**Fig. 11:**
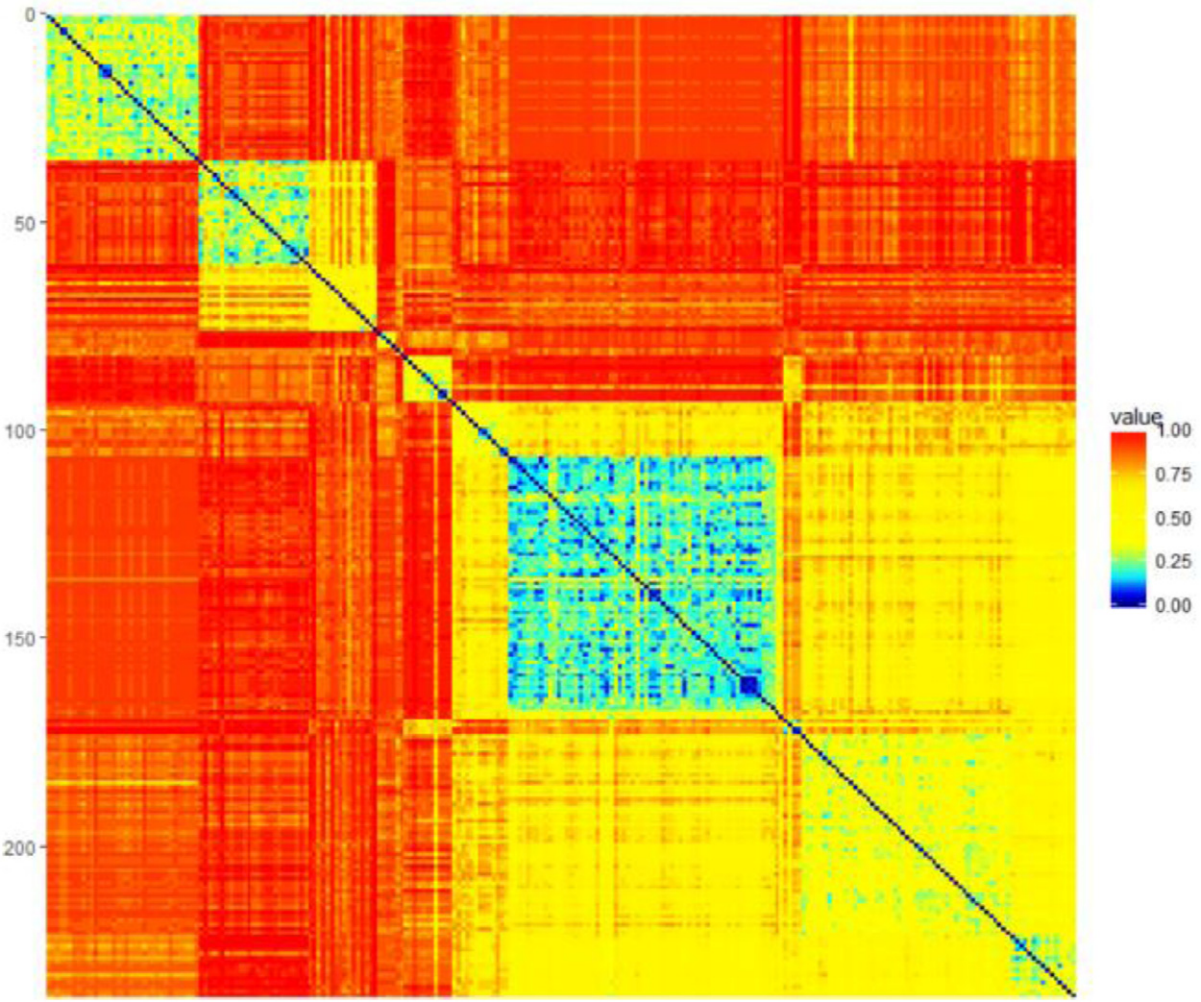
Heatmap of the distances in the Tetragonula dataset. The distances are not sorted. A high-dimensional distance structure is visible. Any clustering should have smaller intracluster than intercluster distances while remaining coherent with the geographic origins.

The raw data are available to the public in the R package prabclus on CRAN: “It contains the genetic data of 236 Tetragonula (Apidae) bees from Australia and Southeast Asia. The data give pairs of alleles (codominant markers) for 13 microsatellite loci. The 13 string variables consist of six digits each” [19]. The format is derived from the data format used by the GENEPOP 4.0 software implemented by Rousset in 2010. “Alleles have a three digit code, so a value of ‘258,260’ on variable V10 means that on locus 10, the two alleles have codes 258 and 260. ‘000’ refers to missing values” [19]. The shared allele distance is described in [20] (p. 493) as follows: “[The distance is] defined as one minus the proportion of alleles shared by 2 individuals averaged over loci. Loci with missing values are not considered in the pairwise distance calculation. In the presence of missing values, this distance measure is not necessarily a metric”. For the distance calculation, the R package fpc of [20] was used, along with the distance introduced by [Bowcock et al., 1994]. The distances are visualized in Fig. 11 as a heatmap.

The geographic origins of the bees saved in “TetragonulaDataSetCoordinates.lrn” are defined as follows: “Longitude (x-axis) and latitude (y-axis) of locations of individuals in decimal format, i.e. one number is latitude (negative values are South), with minutes and seconds converted to fractions. The other number is longitude (negative values are West)” (see [19] and the prabclus package).

### 1.12 Leukemia

The anonymized leukemia dataset consists of 12,692 gene expressions from 554 subjects and is available from a previous publication [21]. The challenge is to find an appropriate clustering w.r.t. to the diagnosis of subjects in the high-dimensional data. Each gene expression is a logarithmic luminance intensity (presence call), which was measured using Affymetrix technology. The presence calls are related to the number of specific RNAs in a cell, which signals how active a specific gene is. Of the subjects, 109 were **healthy**, 15 were diagnosed with acute promyelocytic leukemia (**APL**), 266 had chronic lymphocytic leukemia (**CLL**), and 164 had acute myeloid leukemia (**AML**). “The study design adhered to the tenets of the Declaration of Helsinki and was approved by the ethics committees of the participating institutions before its initiation” [21].

The leukemia dataset was preprocessed, resulting in a high-dimensional dataset with 7747 variables and 554 data points separated into natural clusters, as determined by the illness status and defined by the patterns of change in distance and density. The challenge is to reproduce the highly unbalanced class sizes without ignoring the small APL class by depicting it as noise.

## 2 Experimental design, materials, and methods

The visualizations provided here are generated by the R package ‘DataVisualizations’ available on CRAN [15]. All clustering algorithms used in [1] and the datasets can be found in the R package on CRAN (https://CRAN.R-project.org/package=FCPS). The sample size can be changed for any FCPS dataset using the R function “ClusterChallenge” of the FCPS package.

**Fig. 12:**
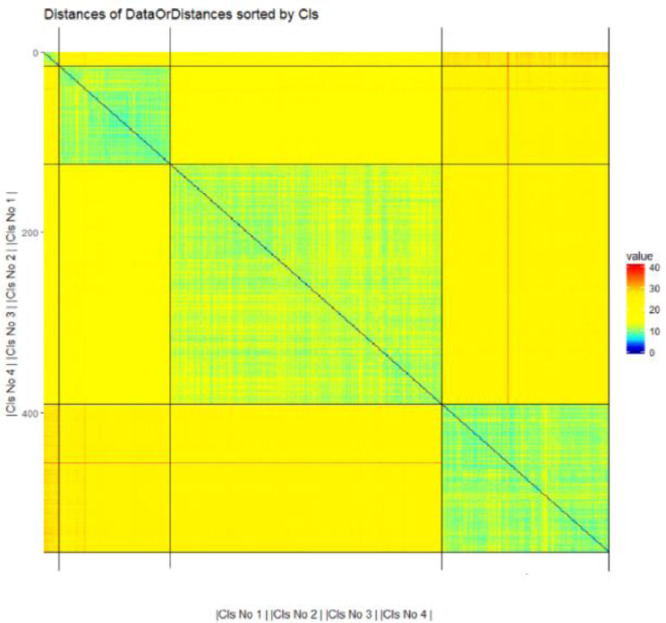
Heatmap of the distances in the Leukemia dataset with four highly unbalanced class sizes. The prior classification defines the order of the distances. The high-dimensional distance structure is defined by the classification and two outliers are visible.

All datasets are also attached to this manuscript and used in [1] to benchmark the clustering algorithms. The DatabionicSwarm clustering used in [11] is provided and visualized in Fig. 12 with the Euclidean distance.
